# Mechanistic View on the Order–Disorder Phase Transition in Amphidynamic Crystals

**DOI:** 10.1021/acs.jpclett.2c03316

**Published:** 2023-02-07

**Authors:** Maor Asher, Marco Bardini, Luca Catalano, Rémy Jouclas, Guillaume Schweicher, Jie Liu, Roman Korobko, Adi Cohen, Yves Geerts, David Beljonne, Omer Yaffe

**Affiliations:** † Department of Chemical and Biological Physics, Weizmann Institute of Science, Rehovot76100, Israel; ‡ Laboratory for Chemistry of Novel Materials, University of Mons, 7000Mons, Belgium; § Laboratoire de Chimie des Polymères, Université Libre de Bruxelles (ULB), 1050Brussels, Belgium; ∥ International Solvay Institutes for Physics and Chemistry, 1050Brussels, Belgium

## Abstract

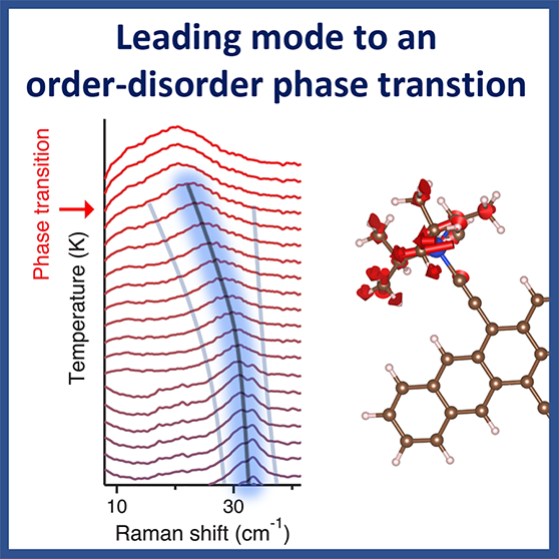

We combine temperature-dependent low-frequency Raman measurements and first-principles calculations to obtain a mechanistic understanding of the order–disorder phase transition of 2,7-di-*tert*-butylbenzo[*b*]benzo[4,5]thieno[2,3*-d*]thiophene (ditBu-BTBT) and 6,13-bis(triisopropylsilylethynyl) pentacene (TIPS-pentacene) semiconducting amphidynamic crystals. We identify the lattice normal modes associated with the phase transition by following the position and width of the Raman peaks with temperature and identifying peaks that exhibit nonlinear dependence toward the phase transition temperature. Our findings are interpreted according to the “hardcore mode” model previously used to describe order–disorder phase transitions in inorganic and hybrid crystals with a Brownian sublattice. Within the framework of this model, ditBu-BTBT exhibits an ideal behavior where only one lattice mode is associated with the phase transition. TIPS-pentacene deviates strongly from the model due to strong interactions between lattice modes. We discuss the origin of the different behaviors and suggest side-chain engineering as a tool to control polymorphism in amphidynamic crystals.

Amphidynamic organic crystals are crystalline materials possessing ordered rigid components linked to mobile elements.^1−4^ The most promising strategies to build amphidynamic crystals are based on crystalline molecular rotors composed of molecules or supermolecular assemblies with two distinct components that can rotate relative to each other (see Figure 1a). One is with a larger moment of inertia which is static (the stator) and another is with a smaller moment of inertia which is rotating (the rotator).^1−6^ As such, amphidynamic crystals are particularly attractive for the design and synthesis of novel molecular functional materials.^2,6−8^ The tunable and switchable relative motion of the components has potential use as actuators, sensors, and shape memory applications.^1,9−11^

**Figure 1:**
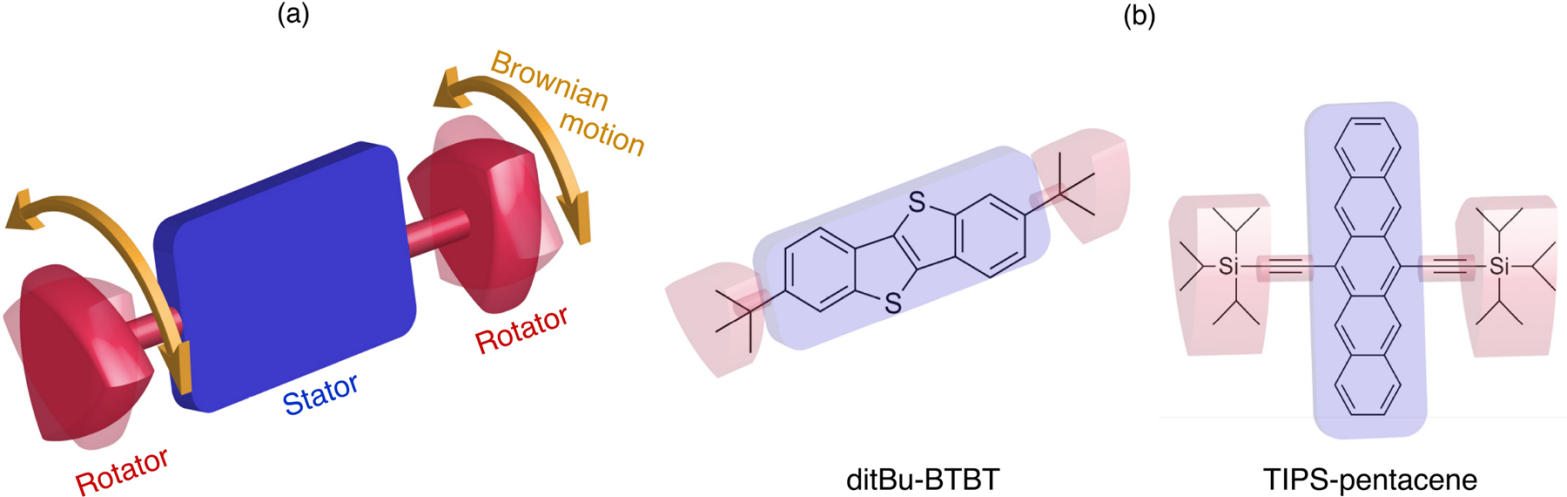
(a) Schematic representation of an amphidynamic molecule. (b) Molecular structures of ditBu-BTBT and TIPS-pentacene, highlighting the stator and rotator components in blue and red, respectively.

Thermal activation of the rotator is often accompanied by a solid–solid phase transition. This phase transition has been experimentally characterized by various methods such as XRD, solid-state NMR, DSC, and high-frequency Raman spectroscopy.^8,11,12^ It is described as a diffusionless, cooperative, martensitic, order–disorder phase transition.^1,11,13^ The mechanism of this phase transition is schematically depicted by a multiwell potential energy surface of the rotator’s rotational motion.^8,11^ Below the phase transition temperature, the rotating component of the molecule is vibrating around the potential minima of one of the wells. Above it, it has enough thermal energy to overcome the rotational energy barrier, resulting in a rotational motion as the atoms jump between equivalent lattice sites (i.e., the disorder mechanism) represented by the bottom of each well.

Such order–disorder phase transitions can be typically described by two modes that are strongly coupled due to the anharmonic nature of the multiwell potential.^14^ The first mode is a vibrational mode that represents the motion at the bottom of each well, and the second represents the motion between the wells, described as a Brownian motion within an intrinsic Brownian sublattice mixed with the ordered lattice.^14−16^ Notably, Andrade and Porto^15,17^ developed a model predicting the temperature evolution of the frequency and width (i.e., lifetime) of the first mode. This model was named “hardcore mode” due to its hardcore frequency at the phase transition temperature, as opposed to the conventional soft mode theory where, at that temperature, its vibrational frequency goes to zero.^17,18^ By fitting the hardcore mode model to experimental data, one can extract the thermal coefficient, which determines the temperature dependence of the potential energy barrier, the correlation time, which is the meantime for the atoms to jump between equivalent lattice sites, and the activation energy, which approximately equals the potential barrier energy.

This method was applied for many inorganic and hybrid molecular crystals that exhibit order–disorder phase transitions.^19−23^ For organic crystals, it was applied only for intramolecular modes and not for lattice modes.^24,25^ For some inorganic and hybrid molecular crystals, the hardcore mode was associated with a single vibrational mode in the material. This implies that in these crystals, there is a single, specific vibrational normal mode that triggers the order–disorder phase transition (similarly to a soft mode that triggers a displacive phase transition^18^). Such triggering by a single vibrational mode has a technological implication that a structural phase transition may be triggered by a resonant electromagnetic pulse.^26−30^ In light of the above, it is interesting to explore if the hardcore model also applies to amphidynamic crystals. In addition, exploring the role of the lattice dynamics on organic crystal polymorphism, which is still poorly understood,^31^ is essential for crystal engineering.

In this study, we compare the temperature evolution of the lattice dynamics across the phase transition of two semiconducting amphidynamic crystals: 2,7-di-*tert*-butylbenzo[*b*]benzo[4,5]thieno[2,3*-d*]thiophene (ditBu-BTBT) and 6,13-bis(triisopropylsilylethynyl) pentacene (TIPS-pentacene)^11,32,33^ (see Figure 1b). We probe their lattice dynamics using temperature-dependent low-frequency (<200 cm^–1^) Raman measurements and assign the peaks to their corresponding eigenvectors through density functional theory (DFT) calculations. The temperature evolution of the Raman spectra is then analyzed according to the hardcore mode model where ditBu-BTBT exhibits ideal behavior. On the contrary, TIPS-pentacene deviates from the model due to strongly coupled (i.e., anharmonic) modes. Finally, we discuss the differences between the order–disorder phase transition mechanisms of ditBu-BTBT and TIPS-pentacene and suggest their physical origin in terms of molecular packing and expressions of vibrational anharmonicity.

Single crystals of ditBu-BTBT and TIPS-pentacene were grown by thermal sublimation and from solution, respectively (see the experimental section for more details regarding the crystals growth procedure). We confirmed the crystal structure and high phase purity of the crystals by performing XRD measurements (see Supporting Information, section S1).

Figure 2a presents the results of the temperature-dependent (80–400 K) low-frequency Raman measurements of ditBu-BTBT. Due to the relatively weak intermolecular interactions in organic solids, this frequency range includes their lattice vibrations (i.e., phonons).^34−37^ Since a hardcore mode is a lattice mode of the system, it is expected to be in this frequency range. Below the phase transition temperature, we see red-shifting and broadening of the spectra as temperature increases. These observations are common in temperature-dependent Raman measurements.^34,38−41^ Their physical origin is mainly thermal expansion which causes a weakening of the intermolecular interaction (red-shifting) and an increase in phonon population, which decreases the vibrational lifetime (broadening).^42^

**Figure 2:**
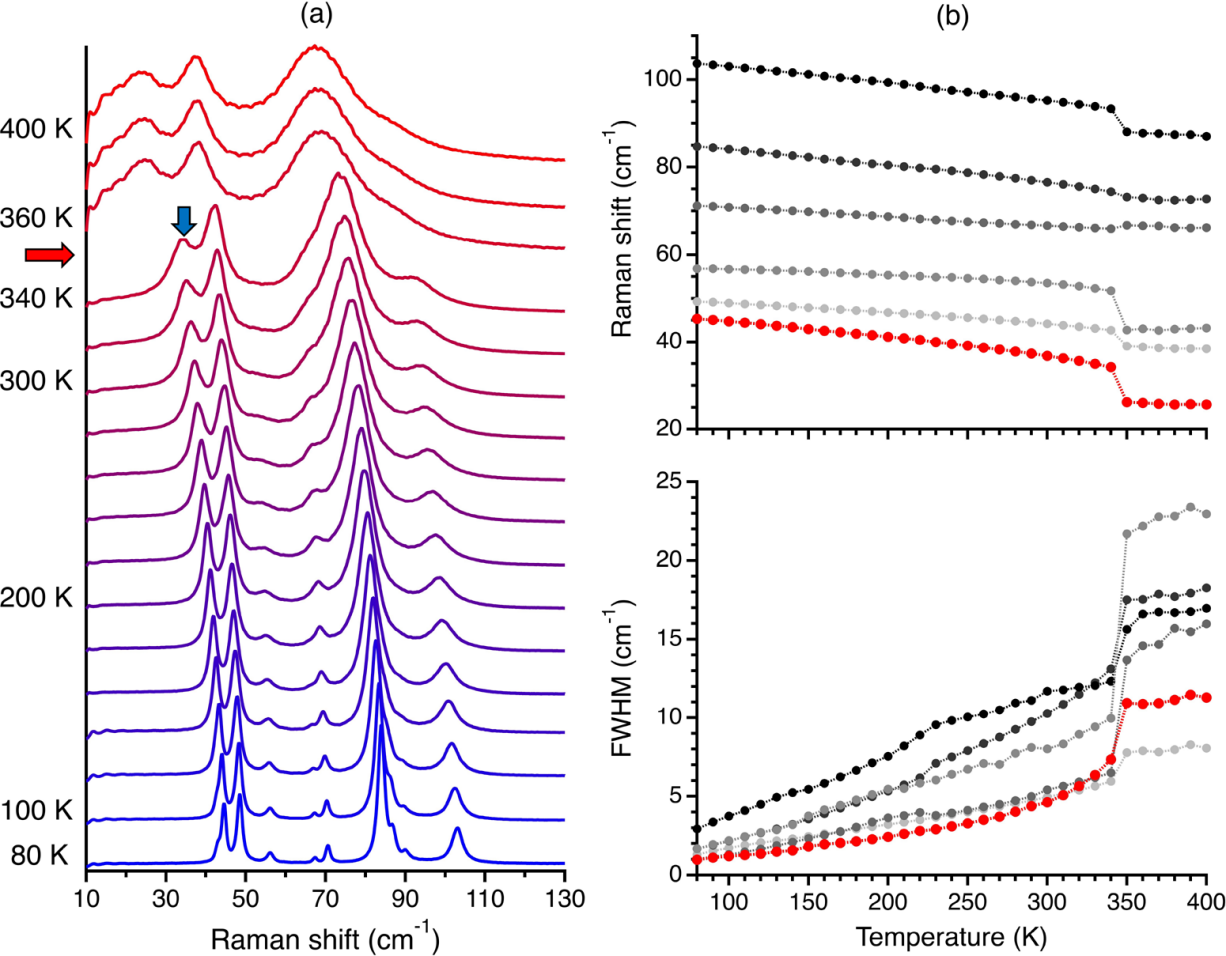
(a) Temperature-dependent low-frequency Raman of ditBu-BTBT (80–400 K). For clarity, we present these results with a temperature increment of 20 K. For all measured Raman spectra in increments of 10 K; see Supporting Information, section S2. The red and blue arrows marked the phase transition temperature and the hard-core mode, respectively. (b) Temperature-dependent vibrational frequencies and fwhm of the lattice vibrations of ditBu-BTBT. The mode we identify as the hardcore mode is in red.

Above the phase transition, we see in Figure 2a the side chains’ rotation effect on the lattice vibrations. As we cross the phase transition temperature, we see an abrupt red-shifting and broadening of the entire Raman spectrum. The phase transition shows a significantly more pronounced response in the low-frequency range compared to the high-frequency range.^11^

Next, we fit each Raman spectrum to the product of the Bose–Einstein distribution and a multidamped Lorentz oscillator (see Supporting Information, section S3, for more details). At each temperature, we measured the Raman spectrum at three polarization angles (0°, 45°, and 90°) to extract the vibrational frequency accurately and fwhm of each mode (see Supporting Information, section S3, for more details). Figure 2b presents the results of this analysis. Several peaks could be resolved only at low temperatures (see Supporting Information, section S3). The fwhm of the lowest-frequency peak changes strongly with temperature compared the all other peaks. The linear correlation coefficient at the low-temperature phase (80–340 K) is above 0.99 for all peaks’ fwhm temperature dependence except for the red mode, which is 0.95. This is the signature of a hardcore mode.^15^ The breakdown of the linear trend of the lowest-frequency peak fwhm temperature dependence is shown clearer in Figure 3b.

**Figure 3:**
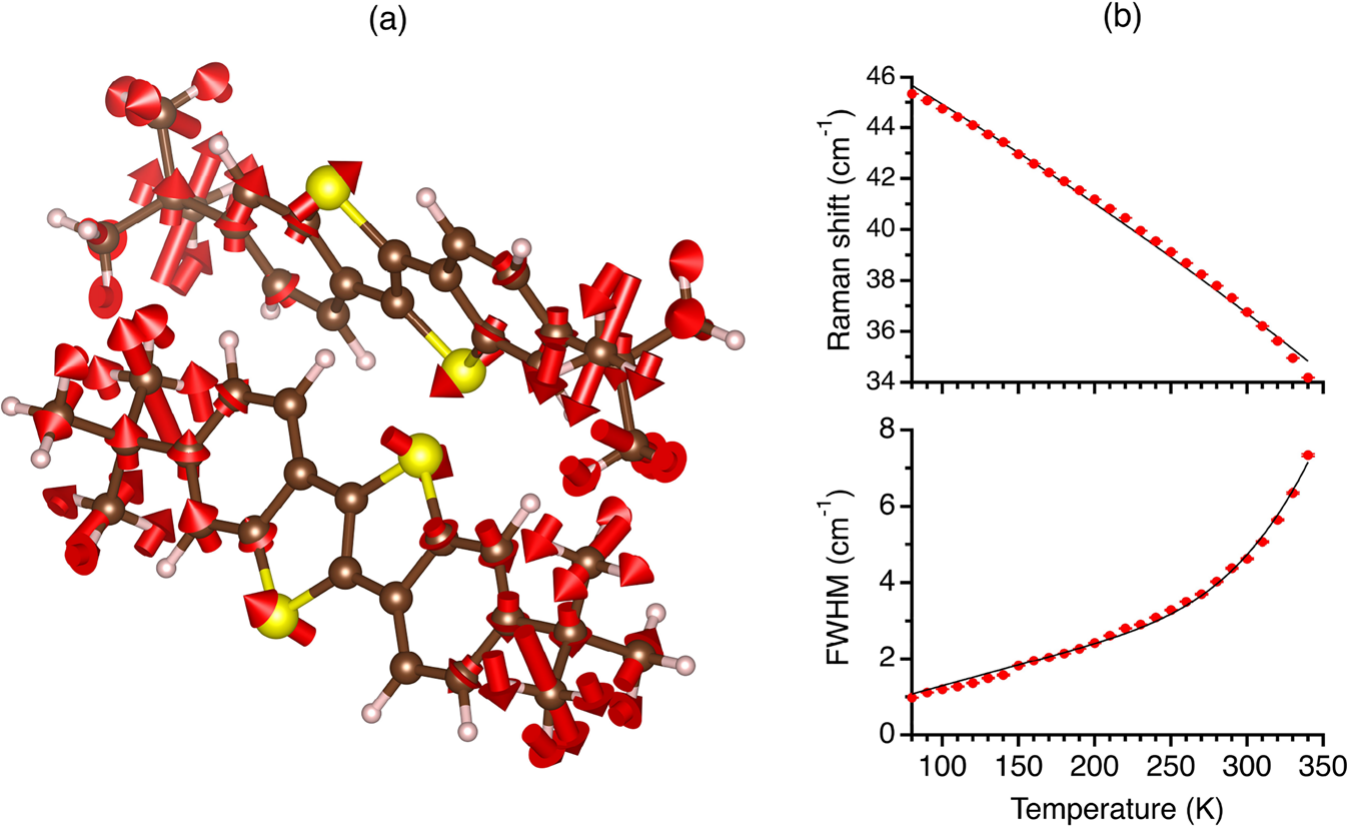
(a) Eigenvector of the hardcore mode of ditBu-BTBT. For clarity, we removed the displacement arrows from atoms with low-amplitude motion, showing this mode includes mainly a torsional motion of the side chains. (b) Fit results for the vibrational frequency and fwhm temperature dependence of the hardcore mode of ditBu-BTBT. The data is shown in solid red circles, including its error bars, and the solid black lines are the fit results to eq 1 and 2

Figure 3a presents the computed eigenvector of the hardcore mode (see Method for more details of the used computational methods and Supporting Information, section S4, for the complete mode assignment). The arrows for atoms with low-amplitude motion were removed for clarity. The eigenvectors and the eigenvectors of the rest of the modes are found in the Supporting Information. We can see that the vibrational motion of the hardcore mode includes mainly torsional motions of the side chains. These are the same side chains that rotate after the phase transition. Hence, these results support that this lattice vibration can be associated with the vibrational motion at the bottom of the multiwell potential energy surface.

Having established that there is only one lattice mode strongly coupled to the phase transition, we now extract the thermal coefficient, correlation time, and activation energy of this mode.^15,17^ The expression for the temperature dependence of the vibrational frequency (ω) of the hardcore mode is
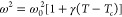
1where *T*_*c*_ is the phase transition temperature, ω_0_ is the vibrational frequency of the hardcore mode at that temperature, and γ is the thermal coefficient. The thermal coefficient determines the variation of the potential energy barrier, assuming a linear thermal expansion.^17^ We fit eq 1 to the experimental data to extract γ. In addition, the expression for the fwhm temperature dependence is^15^
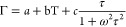
2The first two terms on the right side of eq 2 represent the peak’s broadening due to anharmonic effect (e.g., phonon–phonon interactions), and the third is due to the relaxational motion of the multiwell potential derived by invoking the fluctuation–dissipation theorem.^15^ Accordingly, *a*, *b*, and *c* are fit parameters and τ is the reorientation correlation time,
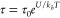
3where *k*_*b*_ is the Boltzmann constant and τ_0_ is usually assumed to obey the Eyring relation,
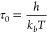
4where *h* is the Planck constant and *U* is the activation energy,
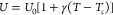
5where *U*_0_ is the activation energy at the phase transition temperature. We use the extracted thermal coefficient and eqs 3–5 in eq 2 to extract *U*_0_ by fitting the experimental fwhm temperature dependence. Studies have shown that the activation energy extracted by eq 2 agrees with other experimental methods.^43−45^

The results of this procedure are presented in Figure 3b for the identified hardcore mode of ditBu-BTBT. There is an excellent fit between the model and experimental results for vibrational frequency and fwhm temperature dependence. We shall see later in the text while discussing TIPS-pentacene that such a good fit is not obvious. The extracted thermal coefficient (γ) and activation energy at the phase transition temperature (*U*_0_) are 2.82 × 10^–3^ ± 5 × 10^–5^ K^–1^ and 2.5 ± 0.2 kcal mol^–1^, respectively. Using eq 3, we calculate a correlation time of 3.7 × 10^–11^ s for the rotational motion at the phase transition temperature. The obtained activation energy is comparable to the DFT-calculated value of 3.3 kcal mol^–1^^46^ and for other amphidynamic crystals.^8,47^

The behavior of ditBu-BTBT concerning the hardcore mode model is a particular case because only one lattice mode in the system is associated with the phase transition. To demonstrate this point, we present the results for TIPS-pentacene, which is also an amphidynamic semiconducting crystal with an order–disorder phase transition.^11,33^Figure 4a shows the results of the temperature-dependent (80–400 K) low-frequency Raman measurements of TIPS-pentacene. The evolution with temperature is more intricate than that of ditBu-BTBT. Below the phase transition (<400 K), we see primarily red-shifting and broadening of the peaks as temperature increases. One exception is the ∼80 cm^–1^ peak of TIPS-pentacene (the peak marked in blue in Figure 4b) that is blue-shifting as temperature increases. This is probably due to the significant quartic anharmonic term known for materials with negative thermal expansion, such as TIPS-pentacene.^33,48,49^ Above the phase transition temperature, we see a weaker effect on the spectrum than ditBu-BTBT, as the abrupt red-shifting and broadening are smaller. Another effect of the phase transition is observed for the ∼80 cm^–1^ peak, which goes from blue-shifting to red-shifting across the phase transition temperature as the temperature increases.

**Figure 4:**
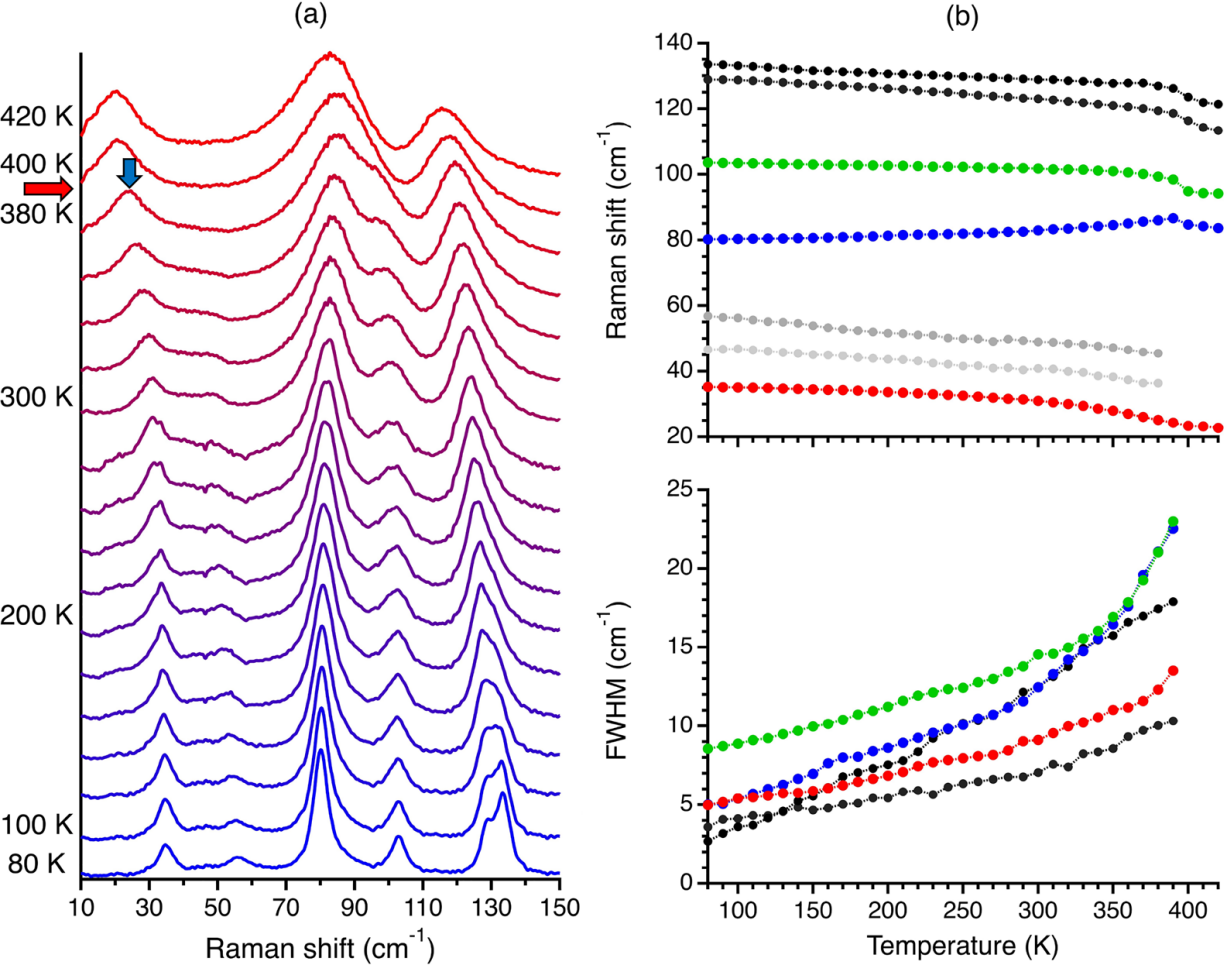
(a) Temperature-dependent low-frequency Raman of TIPS-pentacene (80–400 K). For clarity, we present these results with a temperature increment of 20 K. For all measured Raman spectra in increments of 10 K, see Supporting Information, section S2. The red and blue arrows marked the phase transition temperature and the hardcore mode, respectively. (b) Temperature-dependent vibrational frequencies and fwhm of the lattice vibrations of TIPS-pentacene. The mode we identify as the hardcore mode is colored in red.

Figure 4b presents the extracted temperature dependence of the vibrational frequencies and fwhm of the lattice vibrations. For some of the peaks of TIPS-pentacene, we could not reliably extract the fwhm temperature dependence due to the proximity of the peaks and relatively low intensity. While the peaks marked in grayscale show linear temperature dependence, we observe *three* modes (colored in red, blue, and green), which have a sharper increase in their fwhm temperature dependence while approaching the phase transition temperature (their linear correlation coefficient at the low-temperature phase is below 0.98). This is contrary to the case of ditBu-BTBT, where we observe this hardcore mode-like fwhm temperature dependence for a single peak.

Figure 5 highlights the evolution with temperature of the colored peaks. Notably, their vibrational frequencies also evolve in a nonlinear fashion toward the phase transition temperature. Since the hardcore mode model (eq 1) cannot capture these trends, it is inadequate for TIPS-pentacene, which goes beyond the perturbative treatment of this model.^17^ Hence, the phase transition mechanism in TIPS-pentacene involves the strong coupling of at least three highly anharmonic lattice vibrations.

**Figure 5:**
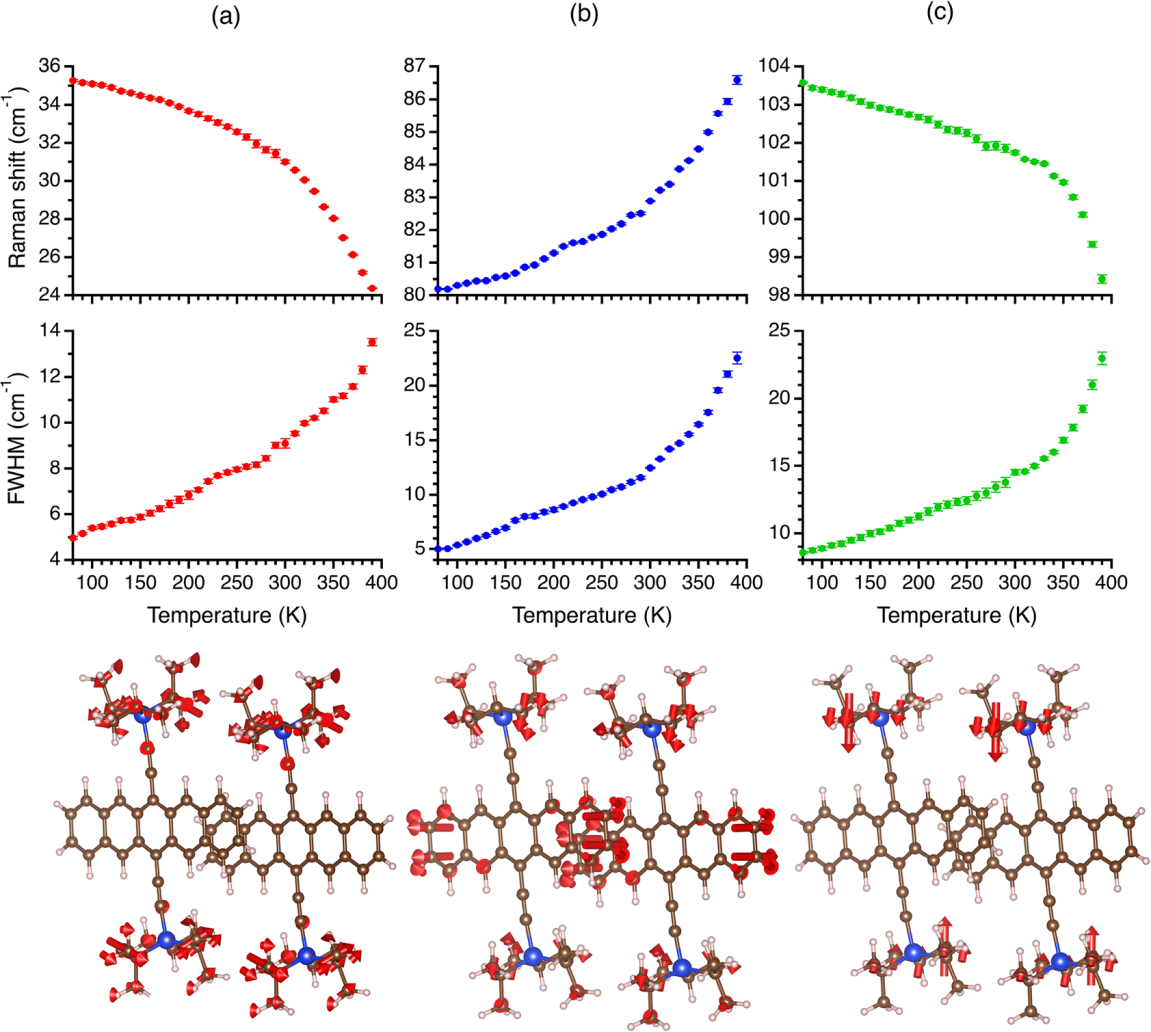
Vibrational frequency (top panel) and fwhm (middle panel) temperature dependence along with their DFT-calculated eigenvectors (bottom panel) of the (a) red, (b) blue, and (c) green mode of TIPS-pentacene from Figure 4. For clarity, we removed the displacement arrows from atoms with low-amplitude motion. The modes include mainly a (a) torsional and (b, c) translational motion of the side chains.

These results indicate that the nonlinear trend in the vibrational frequency and fwhm temperature dependencies toward the phase transition originates not only from the orientational disorder but also from significant anharmonic terms of the potential energy surface, which can also produce similar trends.^50,51^ Notably, the temperature dependence of the vibrational frequency and fwhm depend on the same anharmonic terms of the potential surface. Specifically, the quartic anharmonic term, which is responsible for four-phonon processes, is known to produce such trends.^52^ As mentioned above, the blue shifting of the peak marked in blue in Figure 4b as temperature increases was another indication of a significant quartic term. Furthermore, we suggest that all three colored peaks in Figure 4b are strongly coupled, as the linearity of their vibrational frequency and fwhm temperature dependence break at a similar temperature (around 300 K) far from the phase transition temperature.

The bottom panel of Figure 5 presents the eigenvectors of the three modes that participate in the phase transition. The arrows for atoms with low-amplitude motion were removed for clarity. The eigenvectors and the eigenvectors of the rest of the modes are found in the Supporting Information. While the eigenvector of the mode assigned for the red peak includes mainly a torsional motion of the side chains, the eigenvectors of modes assigned for the blue and green peaks show a different type of motion. The former is dominated by a librational motion of the pentacene backbone, and the latter is dominated by a translational motion of the side chains. These results indicate that in terms of the hardcore model, the motion at the bottom of the potential energy wells includes not only torsional motions of the side chains but other types of motions. These additional motions may play a role in the unlocking of the rotational motion of the side chain by affecting the steric hindrance.

The subtle difference in the order–disorder phase transition mechanism of ditBu-BTBT and TIPS-pentacene may originate from the differences in the molecular structure and crystal packing. As the side chains of TIPS-pentacene include more atoms compared to the side chains of ditBu-BTBT, the TIPS-pentacene molecule has more vibrational degrees of freedom. These are translated to more low-frequency vibrations with a larger role of the side chains motion in the vibrations’ eigenvector, thus increasing their probability of coupling to the rotational motion of the side chains. Another important parameter is the bulkiness of the side chains. Bulkier side chains are known to increase the intermolecular distance and change the molecular packing, loosen the crystal structure, and induce polymorphism.^53^ These effects are intimately related to increasing in vibrational anharmonicity, which is our exact observation in the case of TIPS-pentacene, which has bulkier side chains compared to ditBu-BTBT and shows a more anharmonic behavior.

We show that the order–disorder phase transition mechanism of ditBu-BTBT, an amphidynamic crystal, is associated with a single vibrational mode and exhibits a near-ideal behavior concerning the hardcore mode model. Using this model, we extract the properties of the multiwell potential that represents the side chains’ rotational motion. We contrast the behavior of ditBu-BTBT with that of TIPS-pentacene, where we identify three strongly coupled and anharmonic modes associated with the phase transition. Our results highlight the importance of the molecular structure and crystal packing on the mechanism leading to phase transitions in amphidynamic crystals. This work sheds light on the role of lattice dynamics on molecular crystal polymorphism, paving the way for a rational design of organic crystals undergoing cooperative phase transitions.

## Methods

*Crystals Growth.* ditBu-BTBT single-crystals were grown by thermal sublimation in a Severn Thermal Solutions *TF*50/7.5/3*Z*/*F* furnace at 315 °C with a temperature gradient of −2 K cm^–1^ under argon flow of 0.5 mL min^–1^. TIPS-pentacene single crystals were grown by slow evaporation from an 8 mg mL^–1^ ethyl acetate solution at room temperature.

*Temperature-Dependent Low-Frequency Raman.* A custom-built Raman system was used to conduct the Raman measurements. A 785 nm Toptica diode laser with an intensity of around 30 mW on the sample was used to measure ditBu-BTBT. With this laser, the detection was based on a back-illuminated EMCCD. To avoid photoluminescence and sample heating, a 1064 nm Coherent Nd:YAG solid-state laser with an intensity of around 40 mW on the sample was used to measure TIPS-pentacene. With this laser, the detection was based on a liquid nitrogen-cooled InGaAs detector. To control the polarization of the incident and scattered light for the polarization-dependent measurements, rotating half-wave plates and a polarizer–analyzer combination were used. The system included a 50× objective. Notch filters are included in the system to allow access to the low-frequency region (>10 cm^–1^) and simultaneous acquisition of the Stokes and anti-Stokes signal. The system is based on a 1 m long Horiba FHR-1000 dispersive spectrometer with an 1800 mm^–1^ grating. The spectral resolution was approximately 0.15 cm^–1^. The temperature was set and controlled by a Janis cryostat ST-500 and a temperature controller by Lakeshore model 335.

*DFT Calculations.* Solid-state DFT simulations were performed using the fully periodic CRYSTAL17 software package.^54,55^ The calculations were initiated using the experimental atomic positions and lattice vectors retrieved from Cambridge Crystallographic Data Centre (CCDC). Prior to any vibrational analyses, all atoms were allowed to fully relax with no constraints other than the space group symmetry of the solid and the lattice vectors. Frequency calculations were executed using the optimized coordinates to yield the vibrational modes and Raman intensities. Eigenvalues and eigenvectors were calculated numerically through the harmonic approximation,^56^ and Raman intensities were calculated from the dipole moment derivatives, which were determined using the Berry phase method.^57^ Reciprocal space sampling was performed using the MonkhorstPack scheme, with a *k*-point mesh in the first Brillouin Zone (program keyword SHRINK: X X X). The tolerances for Coulomb and exchange integral cutoffs were set to *ΔE* < 10^–8^ Hartree (program keyword TOLINTEG: 8 8 8 8 16). The energy convergence criterion for geometric optimizations was set to *ΔE* < 10^–12^ Hartree (program keyword TOLDEE: 12). The energy convergence criterion for frequency calculations was likewise set to *ΔE* < 10^–12^ Hartree. The Pople basis set 6-31G*^58,59^ was utilized for all calculations. The GGA class functional Perdew–Burke–Ernzerhof (PBE)^60^ was used for all calculations. London dispersion forces were accounted for using the Grimme DFT-D3 correction.^61^

## Supporting Information Available

Additional results from powder XRD and detailed temperature-dependent Raman measurements, details about the Raman spectra analysis procedure, and DFT calculations results (PDF)
Eigenvectors of the three modes that participate in the phase transition and the eigenvectors of the rest of the modes (ZIP)
Transparent Peer Review report available (PDF)
