# Supplementary material for: Mechanistic View on the Order–Disorder Phase Transition in Amphidynamic Crystals
Source: J Phys Chem Lett. 2023 Feb 7;14(6):1570–7. doi: 10.1021/acs.jpclett.2c03316 (PMC9940296; doi:10.1021/acs.jpclett.2c03316)
Supplement: Supplementary file 1 — jz2c03316_si_001.pdf [file jz2c03316_si_001.pdf]

Supporting Information

A Mechanistic View On The Order-Disorder Phase Transition In Amphidynamic Crystals

Maor Asher,[1] *Marco Bardini,*[2] *Luca Catalano,*[3] *Rémy Jouclas,*[3] *Guillaume Schweicher,*[3] *Jie Liu*[3] *Roman Korobko,*[1]*, Adi Cohen,*[1] *Yves Geerts,*[3,4] *David Beljonne,*[2] *and Omer Yaffe*[1*]

[1]Department of Chemical and Biological Physics Weizmann Institute of Science, Rehovot 76100, Israel.

[2]Laboratory for Chemistry of Novel Materials, University of Mons, 7000 Mons, Belgium

[3]Laboratoire de Chimie des Polymères, Université Libre de Bruxelles (ULB), 1050 Brussels, Belgium

[4]International Solvay Institutes for Physics and Chemistry, 1050 Brussels, Belgium

*omer.yaffe@weizmann.ac.il

S1 Powder X-ray diffraction measurements

Phase purity of ditBu-BTBT and TIPS-pentacene were analyzed using powder X-ray diffraction. All the experiments were performed at room temperature. Compounds were finely ground using a mortar and pestle for phase confirmation measurement. Single crystals were mounted over the sample holder for preferential orientation analysis. For both ditBu-BTBT and TIPS-pentacene, the measurements were conducted on a Panalytical Empyrean diffractometer using Cu–Kα radiation (λ =1.54178 Å). The diffractometer was set up with reflection-transmission spinner 3.0 configuration, and patterns were collected with 2θ range between 5.0 and 30.0°. Calculated patterns were obtained from known crystal structures, ditBu-BTBT [1, 2], and TIPS-pentacene [2] using Powder Pattern tool on Mercury software [3, 4]. The crystalline phase of ditBu-BTBT and TIPS-pentacene were confirmed to have the same phase of the known crystal structures with CSD Refcode KUDFAS01 [2] (ditBu-BTBT), VOQBIM02 (TIPS-pentacene) as it can be observed in Figure S1 (experimental diffraction pattern in red and calculated diffraction patterns in black).

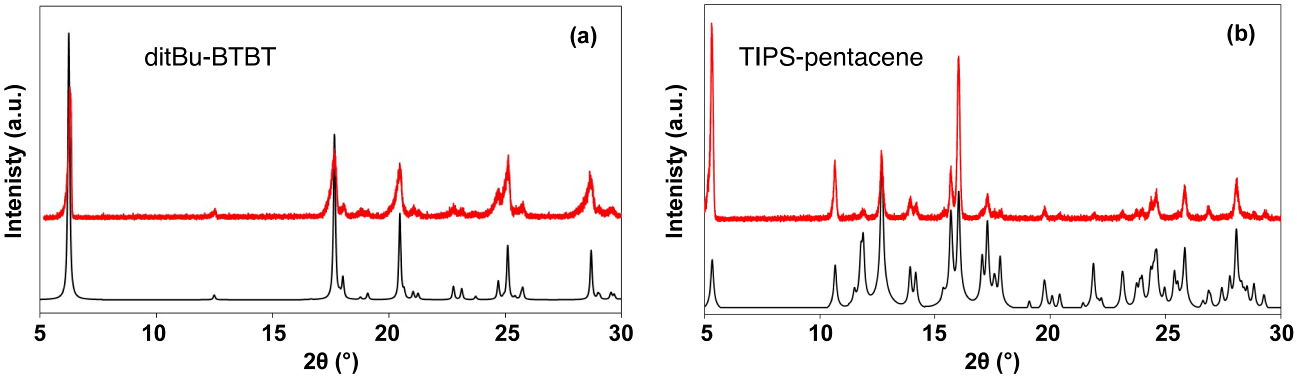

Figure S1: X-Ray diffraction patterns calculated from the known crystal structures (in black), obtained experimentally (in red) (a) ditBu-BTBT and (b) TIPS-pentacene.

S2 Temperature dependent low-frequency Raman spectroscopy

Figure S2 shows the temperature dependent low-frequency Raman spectroscopy spectra of ditBu-BTBT and TIPS-pentacene from 80 K to 400 K at increments of 10 K.

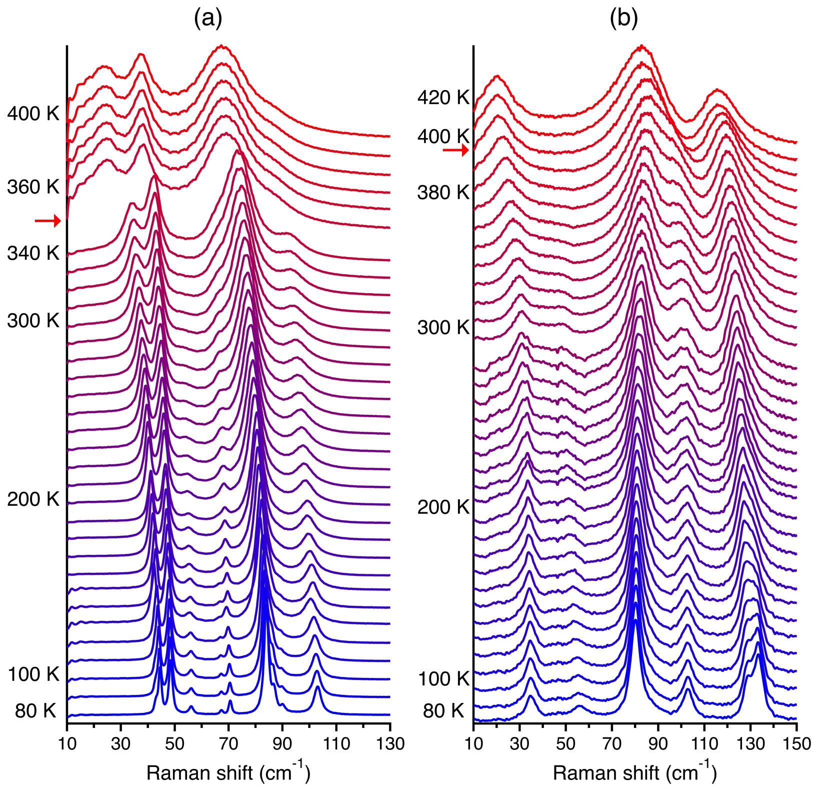

Figure S2: Temperature dependent low-frequency Raman of ditBu-BTBT and TIPS-pentacene. The spectra were normalized and shifted up for clarity. The temperature increment is 10 K. The red arrows indicate the order-disorder phase transition temperature.

S3 Raman spectra fitting

We fit the measured Stokes-shift Raman spectra with the product of the Bose-Einstein distribution and a multi-damped Lorentz oscillator line shape,

$$I_{Raman}(\omega) = \left(\frac{1}{e^{\frac{\hbar\omega}{k_B T}} - 1} + 1 \right) \sum_i \frac{c_i |\omega| \Gamma_i^3}{\omega^2 \Gamma_i^2 + (\omega^2 - \omega_i^2)^2} \tag{1}$$

Where $\omega_{0,i}$, c_i and Γ_i are the position, intensity, and FWHM of each peak, respectively, ω is the measured frequency (Raman shift), T is the temperature, $\hbar$ is the reduced Planck constant and k_B is the Boltzmann constant. The Lorentz in Equation 1 is a variation of the Lorentz oscillator where c is the max value of the peak.

To accurately extract the temperature dependence of the vibrational frequency and FWHM of the lattice vibrations we use the polarization dependency of the low-frequency Raman signal. A half-wave plate determines the linear polarization of the incident laser and a half-wave plate - polarizer (analyzer) combination determines the measured component of the scattered light (for more details about the experimental setup of the polarization-dependent Raman measurements see Ref. [5]. We measure the Raman signal for selected polarization angles where different groups of peaks are most pronounced (see Figure S3). From each of them we extract the vibrational frequencies and FWHMs of the pronounced peaks by fitting the Eq. 1.

Figure S4 presents the fit results for ditBu-BTBT for all low-frequency lattice vibrations, including those which we could resolve only at low temperature (colored in pink).

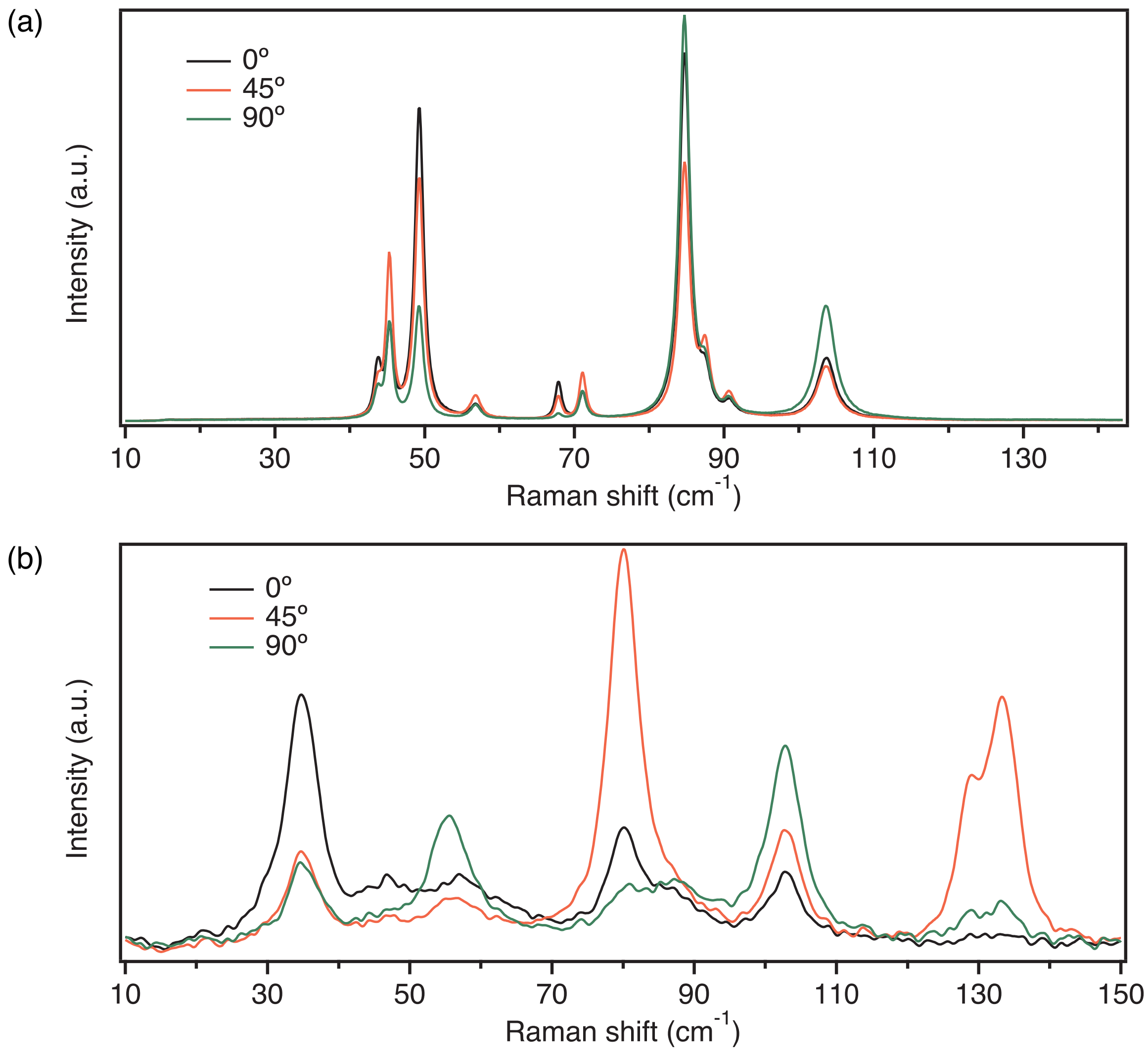

Figure S3: The Raman spectra of (a) ditBu-BTBT and (b) TIPS-pentacene in the parallel configuration at 80 K for selected polarization angles.

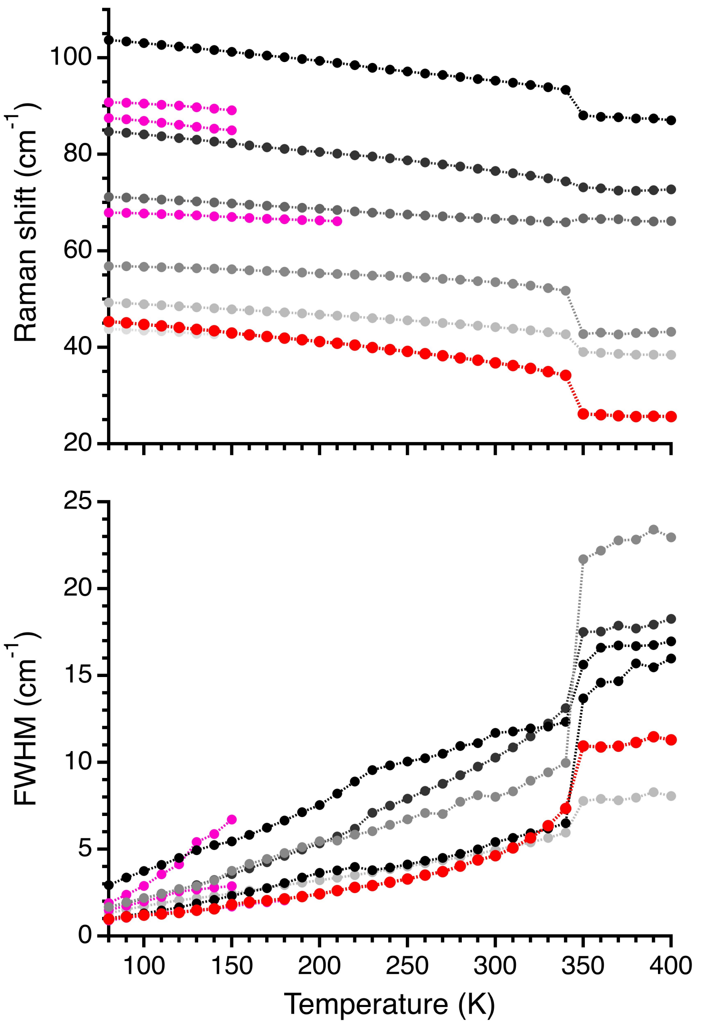

Figure S4: The temperature-dependent vibrational frequencies and FWHMs of the lattice vibrations of ditBu-BTBT.

S4 DFT calculations

Tables S1 and S2 present the mode assignment to the experimental lattice vibration of ditBu-BTBT and TIPS-pentacene, respectively, using the DFT-calculated Raman active modes based on peak proximity and intensity. The vibrational symmetry of ditBu-BTBT were obtained from Ref. [5] and due to the low symmetry of the TIPS-pentacene crystal all lattice vibration have the same vibrational symmetry.

The agreement between the measured and calculated Raman peaks is relatively good. These values are common for this type of calculations [6–9]. The modes eigenvectors for each material are attached to this publication as media (.xsf) files.

Table S1: Mode assignment: The calculated (and experimental) frequency of each of the low-frequency Raman modes of ditBu-BTBT, alongside the calculated (and experimental) symmetry of each mode and its relative intensity. The calculation was performed on a SCXRD structure of ditBu-BTBT performed at 100 K after a full computational relaxation of atomic positions.

	ω_1	ω_2	ω_3	ω_4	ω_5
Freq. (exp) (cm^{-1})	40 (44)	41 (47)	47 (50)	52 (57)	65 (68)
Symmetry (exp)	A_g (A_g)	B_g (B_g)	A_g (A_g)	B_g (B_g)	A_g (A_g)
Rel. Intensity	0.303	0.065	0.181	0.045	0.106
	ω_6	ω_7	ω_8	ω_9	ω_{10}
Freq. (exp) (cm^{-1})	75 (72)	86 (86)	89 (89)	94 (92)	107 (95)
Symmetry (exp)	B_g (B_g)	A_g (A_g)	B_g (B_g)	B_g (B_g)	A_g (A_g)
Rel. Intensity	0.055	1.000	0.028	0.175	0.930

Table S2: Mode assignment: The calculated (and experimental) frequency of each of the low-frequency Raman modes of TIPS-pentacene, alongside the calculated (and experimental) symmetry of each mode and its relative intensity. The calculation was performed on a SCXRD structure of TIPS-pentacene performed at 100 K after a full computational relaxation of atomic positions.

	ω_1	ω_2	ω_3	ω_4	ω_5
Freq. (exp) (cm^{-1})	25 (35)	30	42 (46)	47 (57)	51
Symmetry (exp)	A_g (A_g)	A_g	A_g (A_g)	A_g (A_g)	A_g
Rel. Intensity	0.014	0.025	0.170	0.200	0.049
	ω_6	ω_7	ω_8	ω_9	ω_{10}
Freq. (exp) (cm^{-1})	62 (80)	68 (87)	73	78	84 (103)
Symmetry (exp)	A_g (A_g)	A_g (A_g)	A_g	A_g	A_g (A_g)
Rel. Intensity	0.883	0.050	0.103	0.116	0.711
	ω_{11}	ω_{12}	ω_{13}	ω_{14}	ω_{15}
Freq. (exp) (cm^{-1})	89	107	120 (129)	123 (133)	140
Symmetry (exp)	A_g	A_g	A_g (A_g)	A_g (A_g)	A_g
Rel. Intensity	0.982	0.048	0.458	1.000	0.0 18

References

(1) Schweicher, G. et al. *Advanced Materials* **2015**, *27*, 3066–3072.

(2) Chung, H.; Dudenko, D.; Zhang, F.; D'Avino, G.; Ruzié, C.; Richard, A.; Schweicher, G.; Cornil, J.; Beljonne, D.; Geerts, Y.; Diao, Y. *Nature Communications* **2018**, *9*, 1–12.

(3) Macrae, C. F.; Bruno, I. J.; Chisholm, J. A.; Edgington, P. R.; McCabe, P.; Pidcock, E.; Rodriguez-Monge, L.; Taylor, R.; Van De Streek, J.; Wood, P. A. *Journal of Applied Crystallography* **2008**, *41*, 466–470.

(4) Macrae, C. F.; Edgington, P. R.; McCabe, P.; Pidcock, E.; Shields, G. P.; Taylor, R.; Towler, M.; Van De Streek, J. *Journal of Applied Crystallography* **2006**, *39*, 453–457.

(5) Asher, M.; Jouclas, R.; Bardini, M.; Diskin-posner, Y.; Kahn, N.; Korobko, R.; Kennedy, A. R.; Moraes, L. S. D.; Schweicher, G.; Liu, J.; Beljonne, D.; Geerts, Y.; Yaffe, O. *ACS Materials Au* **2022**, DOI: 10.1021/acsmaterialsau.2c00020.

(6) Asher, M.; Angerer, D.; Korobko, R.; Diskin-Posner, Y.; Egger, D. A.; Yaffe, O. *Advanced Materials* **2020**, *32*, 1908028.

(7) Zaczek, A. J.; Catalano, L.; Naumov, P.; Korter, T. M. *Chemical Science* **2019**, *10*, 1332–1341.

(8) Bedoya-Martínez, N.; Schrode, B.; Jones, A. O. F.; Salzillo, T.; Ruzié, C.; Demitri, N.; Geerts, Y. H.; Venuti, E.; Della Valle, R. G.; Zojer, E.; Resel, R. *The Journal of Physical Chemistry Letters* **2017**, *8*, 3690–3695.

(9) Vener, M. V.; Parashchuk, O. D.; Kharlanov, O. G.; Maslennikov, D. R.; Dominskiy, D. I.; Yu. Chernyshov, I.; Yu. Paraschuk, D.; Yu. Sosorev, A. *Advanced Electronic Materials* **2021**, *7*, 2001281.
