# Supplementary material for: Mechanistic View on the Order–Disorder Phase Transition in Amphidynamic Crystals
Source: J Phys Chem Lett. 2023 Feb 7;14(6):1570–7. doi: 10.1021/acs.jpclett.2c03316 (PMC9940296; doi:10.1021/acs.jpclett.2c03316)
Supplement: Supplementary file 2 — jz2c03316_si_002.pdf [file jz2c03316_si_002.pdf]

jz-2022-03316x.R1

Name: Peer Review Information for "A Mechanistic View On The Order-Disorder Phase Transition In Amphidynamic Crystals"

First Round of Reviewer Comments

Reviewer: 1

Comments to the Author

The authors report on the order-disorder phase transition in amphidynamic crystals from a mechanistic perspective. They use temperature-dependent Raman spectroscopy and first-principles calculations of two representative semiconducting amphidynamic crystals. This study reveals valuable insights into functional dynamics in solid-state materials and this reviewer recommends considering it for publication upon addressing the following minor remarks.

- The definition of amphidynamic crystals in the article takes only rotation into consideration, whereas this class of materials can in principle be associated with other degrees of freedom in the solid state and this should be revised accordingly for clarity. The original formulation of Garcia-Garibay should be considered in that regard of amphidynamic materials as condensed phases that “combine crystalline order and liquid-like dynamics, built with lattice-forming elements linked to components that can undergo fast motion” (Vogelsberg, C. S. & Garcia-Garibay, M. A. Crystalline molecular machines: function, phase order, dimensionality, and composition. Chem. Soc. Rev. 41, 1892–1910 (2012); Liepuoniute, I., Jellen, M. J. & Garcia-Garibay, M. A. Correlated motion and mechanical gearing in amphidynamic crystalline molecular machines. Chem. Sci. 11, 12994–13007 (2020)). Similarly, the statement that “amphidynamic crystals are attractive materials for the design and synthesis of solid-state molecular machines” is confusing and potentially misleading, as these materials commonly feature integrated dynamic components that act as molecular switches and machines and this might need to be clarified in that regard.

- The name of ditBu-BTBT needs to be more accurately defined (i.e., 2,7-di-tert-butylbenzo[b]benzo[4,5]thieno[2,3-d]thiophene) with appropriate formatting (tert, [b] and -d] in italic).

- In their analysis, the authors observe a clear distinction between the behavior of two systems, namely ditBuBTBT and TIPS-pentacene, particularly from the perspective of whether the “hardcore mode” model applies to their order-disorder phase transitions. However, while they suggest that this difference in the mechanism emerges from the molecular structure and the corresponding crystal packing, there is no comment on how they actually differ in the solid state. Even though these are known compounds, for a better understanding, it would be important to comment on the differences in their molecular and crystal structure that motivated the authors to focus on these representative model systems. This is further relevant in the context of their conclusion which implies that this work is relevant for the rational design of organic crystals.

- The language is clear and coherent throughout the manuscript. On a minor note, the authors should introduce abbreviations upon their first use for clarity, including those used in the abstract. Moreover, the manuscript should be revised once more for typos (e.g. “teampreture” on page 2) and Figure references should be consistent (e.g. Figure vs. Fig.).

- The literature is appropriate throughout the manuscript and other pioneering work on the topic should be included (such as the work of Garcia-Garibay indicated previously).

Thank you for your consideration.

Reviewer: 2

Comments to the Author

1. What is the major advance reported in the paper?

The manuscript by Asher and co-workers presents an application of the "hard-core" mode model, developed in the 1970s to model order-disorder transitions in dielectric crystals, to amphidynamic crystals with both rigid (stator) and reorientating (rotator) components. The authors show that some systems (di-tBu-BTBT) can indeed be very well described by a single hard-core mode, while in other cases (TIPS-pentacene) multiple coupled modes display hard-core behaviour.

2. What is the immediate significance of this advance?

This will certainly prompt analysis of many more amphidynamic systems in these terms; indeed I suspect that this will soon become a standard analysis method in the field. More generally this raises a very tricky point of crystal engineering: how to develop materials with specific mode behaviour at the order-disorder transition. The methodology developed here will be a powerful tool to support such efforts (although a general solution to this problem is still very far away).

3. Technical suggestions

This is a well-written, interesting, and scientifically sound paper and the following are only very minor suggestions.

The abstract and introduction mention "side-chain engineering" but this is not taken up again in the body of the letter; I would be interested to hear the authors' thoughts on this in the conclusions, but otherwise this should be removed from the start of the paper.

The linear correlation coefficient as signature of a hardcore mode (p2): it is more convincing to show that the hardcore mode *is* well fitted by the extra Lorentzian term than just that it is *not* well fitted by a linear model. The authors show exactly this later on (Fig. 3b) and a reference to this here might help the reader. (That is, it is only clear later, on reading about TIPS-pentacene, why the authors have chosen a linear fit as a preliminary analysis.)

Fig 3a is difficult to interpret. I appreciate the difficulties with displaying this sort of diagram and I am sure that the authors have chosen the best orientation possible, but it might also be helpful to describe the motion in words in the figure caption (specifically what the tert-butyl groups are doing: are those methyl rotations or something more complicated?).

S4, DFT: there is a slight contradiction between the Methods section and SI in that the Methods say that the atomic positions and cell vectors were optimised while the SI says that only the atomic positions were. I suspect this is just an omission in SI but it should be clarified. For completeness, the temperatures of the initial SCXRD data should also be given.

Author's Response to Peer Review Comments:

Omer Yaffe
Dep. of Chemical & Biological Physics
Weizmann Institute of Science
Rehovot, Israel
+97289343979
E-mail: omer.yaffe@weizmann.ac.il

December 20, 2022

To: Editor, JPC Letters

Re: Revision of manuscript ID jz-2022-03316x by M. Asher *et al.*

Dear editor,

On behalf of Prof. Geerts, Prof. Beljonne, and myself, I would like to express our sincere gratitude for the time and effort invested in our manuscript.

The revised version of the manuscript and supporting information contain several edits and additional information that addresses all of the reviewer's comments (see attached point-by-point response). A significant change was made in the conclusion, where we added a paragraph discussing the effect of the molecular structure on the order-disorder phase transition mechanism. The paragraph addresses the main comment shared by both reviewers.

Therefore, we believe that the current version is clearer and that our study merits publication in *JPC Letters*.

Sincerely,

Omer Yaffe, Ph.D.

Point-by-point response

Reviewer 1:

1. Reviewer 1 wrote: The authors report on the order-disorder phase transition in amphidynamic crystals from a mechanistic perspective. They use temperature-dependent Raman spectroscopy and first-principles calculations of two representative semiconducting amphidynamic crystals. This study reveals valuable insights into functional dynamics in solid-state materials and this reviewer recommends considering it for publication upon addressing the following minor remarks.

   We thank reviewer 1 for their comments and positive feedback. We performed the requested revisions as presented below.

2. Reviewer 1 wrote: The definition of amphidynamic crystals in the article takes only rotation into consideration, whereas this class of materials can in principle be associated with other degrees of freedom in the solid state and this should be revised accordingly for clarity. The original formulation of Garcia-Garibay should be considered in that regard of amphidynamic materials as condensed phases that "combine crystalline order and liquid-like dynamics, built with lattice-forming elements linked to components that can undergo fast motion" (Vogelsberg, C. S. & Garcia-Garibay, M. A. Crystalline molecular machines: function, phase order, dimensionality, and composition. Chem. Soc. Rev. 41, 1892–1910 (2012); Liepuoniute, I., Jellen, M. J. & Garcia-Garibay, M. A. Correlated motion and mechanical gearing in amphidynamic crystalline molecular machines. Chem. Sci. 11, 12994–13007 (2020)). Similarly, the statement that "amphidynamic crystals are attractive materials for the design and synthesis of solid-state molecular machines" is confusing and potentially misleading, as these materials commonly feature integrated dynamic components that act as molecular switches and machines and this might need to be clarified in that regard.

   We thank the reviewer for their comment. We revised the manuscript to generalize the definition of amphidynamic crystals and to clarify the role of this class of crystalline systems in building novel functional materials.

   Amphidynamic organic crystals are **crystalline materials possessing ordered rigid components linked to mobile elements.**[1-4] **The most promising strategies to build amphidynamic crystals are based on crystalline molecular rotors composed of** molecules or **supermolecular** assemblies with two distinct components that can rotate relative to each other (see Figure 1a). One with a larger moment of inertia which is static (the stator), and another with a smaller moment of inertia which is rotating (the rotator)[1–6]. As such, amphidynamic crystals are **particularly attractive** for the design and synthesis of **novel molecular functional materials**[4,7,8].

   New References: Ref 3) Vogelsberg, C. S.; Garcia-Garibay, M. A. Crystalline molecular machines: function, phase order, dimensionality, and composition. Chemical Society Reviews 2012, 41,1892-1910. Ref 4) Liepuo-

niute, I.; Jellen, M. J.; Garcia-Garibay, M. A. Correlated motion and mechanical gearing in amphidynamic crystalline molecular machines. Chemical Science 2020, 11, 12994-13007.

3. Reviewer 1 wrote: The name of ditBu-BTBT needs to be more accurately defined (i.e., 2,7-di-tert-butylbenzo [b]benzo[4,5]thieno[2,3-d]thiophene) with appropriate formatting (tert, [b] and -d] in italic).

   We added defined ditBu-BTBT and suggested by the reviewer:

   In this study we compare the teampreture evolution of the lattice dynamics across the phase transition of two semiconducting amphidynamic crystals: **2,7-di-tert-butylbenzo[*b*]benzo[4,5]thieno[2,3-*d*]thiophene** (ditBu-BTBT)

4. Reviewer 1 wrote: In their analysis, the authors observe a clear distinction between the behavior of two systems, namely ditBuBTBT and TIPS-pentacene, particularly from the perspective of whether the "hardcore mode" model applies to their order-disorder phase transitions. However, while they suggest that this difference in the mechanism emerges from the molecular structure and the corresponding crystal packing, there is no comment on how they actually differ in the solid state. Even though these are known compounds, for a better understanding, it would be important to comment on the differences in their molecular and crystal structure that motivated the authors to focus on these representative model systems. This is further relevant in the context of their conclusion which implies that this work is relevant for the rational design of organic crystals.

   We added a paragraph before the conclusion to address the reviewer's comment:

   The subtle difference in the order-disorder phase transition mechanism of ditBu-BTBT and TIPS-pentacene may originate from the differences in the molecular structure and crystal packing. As the side chains of TIPS-pentacene include more atoms compared to the side chains of ditBu-BTBT, the TIPS-pentacene molecule has more vibrational degrees of freedom. These are translated to more low-frequency vibrations with a larger role of the side chains motion in the vibrations' eigenvector, thus, increasing their probability of coupling to the rotational motion of the side chains. Another important parameter is the bulkiness of the side chains. Bulkier side chains are known to increase the intermolecular distance and change the molecular packing, loosen the crystal structure, and induce polymorphism[53]. These effects are intimately related to increasing in vibrational anharmonicity, which is our exact observation in the case of TIPS-pentacene, which has bulkier side chains compared to ditBu-BTBT and shows a more anharmonic behavior.

5. Reviewer 1 wrote: The language is clear and coherent throughout the manuscript. On a minor note, the authors should introduce abbreviations upon their first use for clarity, including those used in the abstract. Moreover, the manuscript should be revised once more for typos (e.g. "teampreture" on page 2) and Figure references should be consistent (e.g. Figure vs. Fig.).

We introduce the abbreviations of ditBu-BTBT and TIPS-pentacene in the abstract, we corrected several typos, and the figure references are now consistently "Figure".

6. Reviewer 1 wrote: The literature is appropriate throughout the manuscript and other pioneering work on the topic should be included (such as the work of Garcia-Garibay indicated previously). Thank you for your consideration.

   The works of Garcia-Garibay were added to the literature and were cited accordingly. See bullet point 2 above.

Reviewer 2:

1. Reviewer 2 wrote: The manuscript by Asher and co-workers presents an application of the "hard-core" mode model, developed in the 1970s to model order-disorder transitions in dielectric crystals, to amphidynamic crystals with both rigid (stator) and reorientating (rotator) components. The authors show that some systems (di-tBu-BTBT) can indeed be very well described by a single hard-core mode, while in other cases (TIPS-pentacene) multiple coupled modes display hard-core behaviour. This will certainly prompt analysis of many more amphidynamic systems in these terms; indeed I suspect that this will soon become a standard analysis method in the field. More generally this raises a very tricky point of crystal engineering: how to develop materials with specific mode behaviour at the order-disorder transition. The methodology developed here will be a powerful tool to support such efforts (although a general solution to this problem is still very far away).

   We are glad reviewer 2 appreciates our manuscript's importance and impact on the fields of amphidynamic crystals and crystal engineering.

2. Reviewer 2 wrote: This is a well-written, interesting, and scientifically sound paper and the following are only very minor suggestions. The abstract and introduction mention "side-chain engineering" but this is not taken up again in the body of the letter; I would be interested to hear the authors' thoughts on this in the conclusions, but otherwise this should be removed from the start of the paper.

   The paragraph we added in bullet point 4 in our response to reviewer 1 addresses this issue raised by reviewer 2.

3. Reviewer 2 wrote: The linear correlation coefficient as signature of a hardcore mode (p2): it is more convincing to show that the hardcore mode *is* well fitted by the extra Lorentzian term than just that it is *not* well fitted by a linear model. The authors show exactly this later on (Fig. 3b) and a reference to this here might help the reader. (That is, it is only clear later, on reading about TIPS-pentacene, why the authors have chosen a linear fit as a preliminary analysis.)

   According to the comment of reviewer 2, we added page 2 the following sentence, referring to Fig. 3b.

   The breakdown of the linear trend of the lowest-frequency peak FWHM temperature dependence is shown clearer in Figure 3b.

4. Reviewer 2 wrote: Fig 3a is difficult to interpret. I appreciate the difficulties with displaying this sort of diagram and I am sure that the authors have chosen the best orientation possible, but it might also be helpful to describe the motion in words in the figure caption (specifically what the tert-butyl groups are doing: are those methyl rotations or something more complicated?).

   We added to the captions of Figure 3 and Figure 5 a description of the motion of each eigenvector.

To the caption of Figure 3: showing this mode includes mainly a torsional motion of the side chains. To the caption of Figure 5: The modes include mainly a (a) torsional and (b,c) translational motion of the side chains.

5. Reviewer 2 wrote: S4, DFT: there is a slight contradiction between the Methods section and SI in that the Methods say that the atomic positions and cell vectors were optimised while the SI says that only the atomic positions were. I suspect this is just an omission in SI but it should be clarified. For completeness, the temperatures of the initial SCXRD data should also be given.

   For the DFT calculations, only the atomic positions were optimized. we corrected the Methods section accordingly by deleting this part to make this paragraph clearer:

   All structures underwent geometry optimization...

   In addition, we added to the caption of the mode assignment tables in the SI that the SCXRD measurements were performed the 100 K.
